# Nurses’ contribution to antimicrobial stewardship: business as usual?

**DOI:** 10.1186/s13756-024-01451-z

**Published:** 2024-08-29

**Authors:** Maria Bos, Cindy de Bot, Hester Vermeulen, Marlies Hulscher, Jeroen Schouten

**Affiliations:** 1 School of Social Work and Health, Avans University of Applied Sciences, ’s Hertogenbosch, The Netherlands; 2 School of Social Work, Education and Health, Avans University of Applied Sciences, Breda, The Netherlands; 3 IQ Health Science Department, Radboud University Medical Center, Nijmegen, The Netherlands; 4 School of Health, HAN University of Applied Sciences, Nijmegen, The Netherlands; 5 Department of Intensive Care Medicine, Radboud University Medical Center, Nijmegen, The Netherlands

**Keywords:** Antimicrobial stewardship, Nurse’s role, Interprofessional collaboration, Leadership, Antibiotic use, Antibacterial agents, Infectious disease, Qualitative research

## Abstract

**Background:**

Antimicrobial Stewardship (AMS), the set of actions to ensure appropriate antimicrobial use, is increasingly considered a multidisciplinary endeavour. However, it is unclear how Dutch hospital-based nurses envision their contribution to AMS.

**Objective:**

To explore the views and visions of Dutch bedside nurses on their role regarding appropriate antimicrobial use.

**Methods:**

A qualitative study using semi-structured interviews was conducted. Fourteen bedside nurses in nine different Dutch hospitals participated. Data were analysed using a thematic content analysis.

**Results:**

Nurses considered their role regarding appropriate use of antibiotics as an integral part of their daily nursing practice. They envisioned their future role as an expansion of their current practice, improving or intensifying this contribution. Prompting review of antimicrobial treatment by nurses was seen as regular practice. Ward rounds were considered the best moment to exert their nursing role, by showing leadership in communicating about different aspects of AMS. Patient advocacy (“striving for the best possible care for their patient”) appears to be a driver of the nursing contribution. Nurses perceived a shared responsibility with prescribers on certain aspects of the antimicrobial treatment and wished for a clarification of this role. Education and cognitive reminders such as antibiotic checklist to be used in ward rounds, can support the uptake of the nurses’ role.

**Conclusion:**

Nurses envision their future role in AMS as an enhanced, elaborated and empowered version of their current daily practice. Education, formal acknowledgment and increased awareness of the nursing role, may advance the contributing role nurses already have.

**Supplementary Information:**

The online version contains supplementary material available at 10.1186/s13756-024-01451-z.

## Introduction

Antimicrobial resistance (AMR), or resistance of micro-organisms to antibiotics, is an increasing global health threat that complicates the treatment of infections and leads to increased mortality, morbidity, length of hospital stay and healthcare cost [1, 2].

Since one of the main drivers of AMR is exposure to antibiotics, a crucial strategy to mitigate AMR is the optimisation of antimicrobial use, as described in the World Health Organisation (WHO) Global Action plan [1]. This optimisation can be reached by applying Antimicrobial Stewardship (AMS), the ‘coherent set of actions which promote using antimicrobials responsibly” [3]. Although Antimicrobial Stewardship programs mainly focus on the traditional professionals involved in prescribing –infectious disease (ID) specialists, medical microbiologists and clinical pharmacists– increasing attention is given to other healthcare professionals, such as nurses, who are involved in the care of patients requiring antimicrobial therapy [4]. This interprofessional approach was already promoted in 2010 by Charani et al., who suggested a whole-system approach to address AMR [4].

Since 2017, the central role of nurses is described in international position statements and reports, such as the Position Statement on Antimicrobial Resistance by the International Council of Nurses (ICN), in reports of the European Federation of Nurses (EFN), American Nursing Association (ANA) and the Australian Commission on Safety and Quality of Health Care (ACSQH) [5–8]. Many nursing activities influencing antimicrobial use can be distinguished, such as the administration of antimicrobials, monitoring of patients while on antimicrobial therapy, collecting specimen for cultures or preventing the transmission of Multidrug resistant micro-organisms (MDROs). Additionally, nurses can participate in multidisciplinary care meetings where intravenous-to-oral switch of antimicrobials is discussed, can assess and discuss prerequisites for Outpatient Antimicrobial Therapy (OPAT), inform patients and family/caregivers about the prescribed course of antibiotics and educate them on AMR in general [5, 7–9].

A recent review [10] found that nurses already perform most of these activities, driven by the motivation to provide the best possible care for their patients. However, it is unknown how bedside nurses in the Netherlands view this professional nursing role.

The aim of this study is therefore to explore the views and visions of Dutch hospital-based bedside nurses on their role regarding appropriate antibiotic use.

## Method

### Study design

We used a qualitative exploratory design using semi-structured interviews. Qualitative designs involve broad research questions that capture the various dimensions of a new situation or event, enabling researchers to present a holistic and rich description [11, 12].The consolidated criteria for reporting qualitative research (COREQ) checklist was used as guidance to report our research findings [13].

### Participants

To gain an overall perspective of nurses‘ views and visions, we applied a purposive sampling strategy, recruiting nurses from different educational backgrounds, settings and professional experience.

Participants were eligible if they were licensed as a registered nurse, worked more than 28 h as a nurse on a surgical or internal medicine ward in a hospital and were able to participate in an interview using the web-based platform Zoom. Nurses who were members of the hospital’s AMS team were excluded.

Nurses were recruited through professional nursing networks and collaborating partners. An eligible participant was invited by a member of such a network of collaboration. If they were potentially willing to participate, they received an email message containing information about the aim and purpose of the research study, details about the timeframe and an informed consent form. After two weeks, the first author contacted the potential participant through email. After four weeks, a follow-up mail was sent as reminder. Those who did not respond to both emails were considered not willing to participate. If willing to participate, nurses could either sign the informed consent form and return it by email or give a verbal consent prior to the interview which was then audiorecorded and separately stored. According to Dutch law, this is deemed sufficient as proof of voluntary participation.

Participants were asked to choose a date and time at their convenience to facilitate participation and equally allow participants who work irregular shifts to participate.

### Data collection

Data were collected through semi-structured interviews using a literature based topiclist (additional file 1) which was thoroughly discussed with the research team and pilot tested with 3 hospital-based bedside nurses. Interviews were conducted by the first author (MB), a female researcher with a background in Intensive Care Nursing and Infection Prevention, working as a lecturer in Nursing Science. The interviewer is trained in qualitative research methods. There was no prior relationship between interviewer and participants.

At the start of the interview, permission was (again) asked to audiotape the interview. The interview started with obtaining demographic information after which the interview proceeded using the topiclist. Interviews were recorded with the participants’ permission and the anonymized recordings were subsequently transcribed verbatim by a professional transcription service. Transcripts were not returned to the participants, but during the interview, findings were summarized and discussed with the participants. No repeat interviews were conducted.

### Data analysis

The transcripts of the interviews were analysed using an inductive thematic analysis approach, as described by Braun & Clark [14]. The analysis was aided by using the software program Atlas.ti version 22. After the first phase of inductive coding, axial coding was initiated followed by thematic coding. All transcripts were coded by 2 researchers (MB/CB). The coding process was an iterative cyclic process, with frequent reflection and discussion of interview and analytic processes. After six interviews, codes and evolving themes were discussed with a third researcher (JS). After 12 analysed interviews, codes, axial codes and themes were discussed with the entire research team (MB/CB/MH/HV/JS). Data saturation was defined as the point where no additional information on the topic was generated [15], which was discussed with the research team.

## Results

### Demographics

Between March and October 2022, we conducted semi-structured interviews with 14 nurses from nine different hospitals in the Netherlands. Nurses had an average of 13.2 years of professional experience and were mostly educated at Bachelor of Science level. Average duration of the interviews was 45 min (range 29–60 min). For further details on characteristics of the participants, see Table 1. We reached data saturation after 12 interviews, which was confirmed by two additional interviews (in total 14 interviews).

**Table 1:** Demographic information participants (*n* = 14)

	*n*(%)
**Gender, female**	13 (92.3)
**Age category (years)**	
20–24 25–29 30–34 35–39 40–45 46–55 55–65	1 (7.1) 6 (42.8) 2 (14.3) 1 ( 7.1) 1 (7.1) 2 (14.2) 1 ( 7.1)
**Educational level (highest, combination possible)**	
Vocational college Bachelor of Nursing Master of Science in Nursing Specialty training, e.g. ICU/Oncology	2 (14.3) 10 (71.4) 2 (14.3) 4 (28.6)
**Nursing experience** in years (average, range)	13.2 (2–37)
**Working in Department, specialty**	
Surgical Internal medicine Combination surgical/internal medicine	7 (50) 6 (42.9) 1 (7.2)
**Hospital category, number of participants**	
Academic medical center Teaching hospital General hospital	4 (28.6) 7 (50) 3 (21.4)

**Fig. 1:**
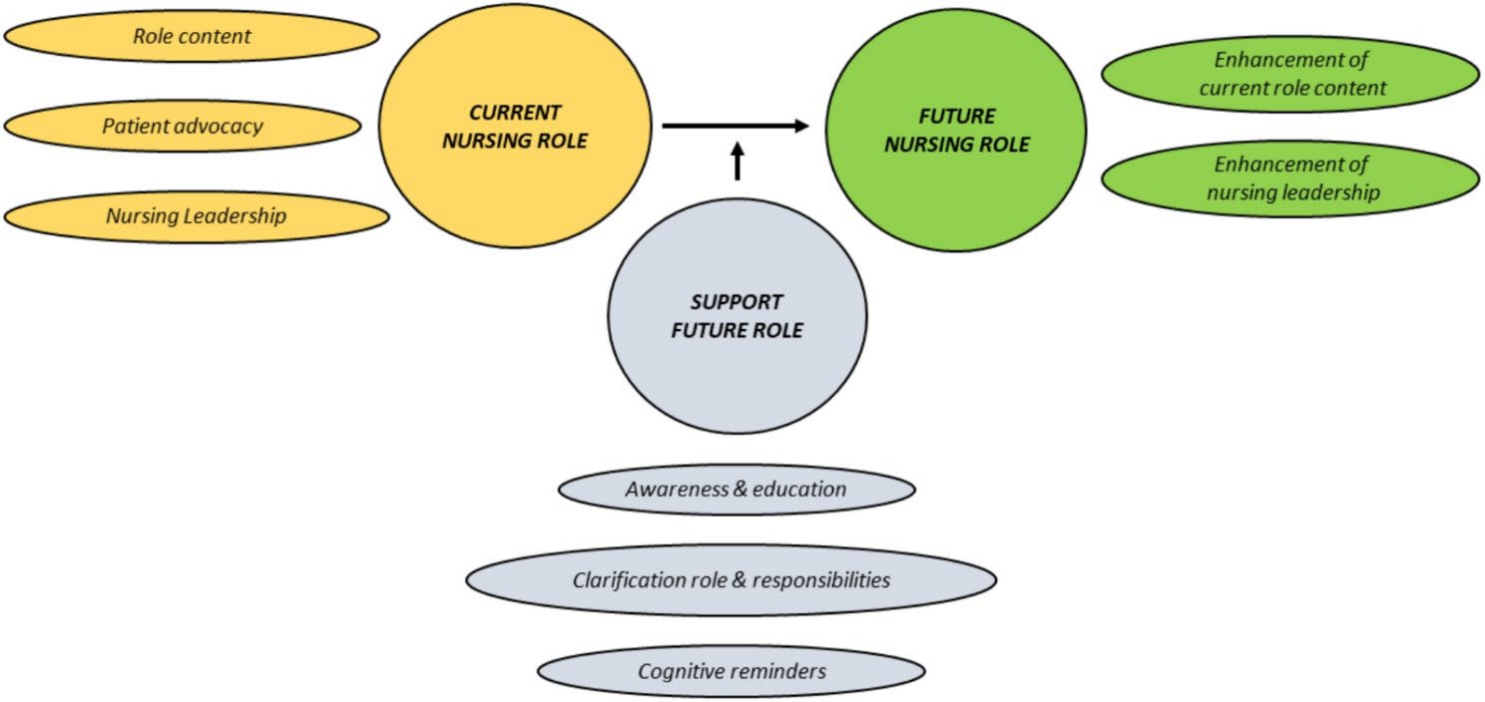
Themes

The following themes were gathered from the responses of nurses: nurses’ current role (role content, patient advocacy and nurse leadership), nurses’ future role (enhanced nurse leadership and enhanced role content ) and the third domain encompasses the building blocks for strengthening nurses’ future role (Fig. 1).

### Nurses’ current role

#### Content of nursing role

When asked about their role as a nurse with regard to appropriate antimicrobial use, all nurses initially explained their role in day-to-day activities related to the antimicrobial treatment. They described that prior or during antimicrobial treatment they assessed the clinical status of a patient and acted upon this, communicating with the physician about potential directions of care. Several nurses considered themselves as a continuous factor in the patient clinical pathway, being able to see changes in patient clinical status on a day-to-day basis.

*…Yes*,* as a doctor it can be very nice to be able to rely on that nurse who was been taking care of the patient for the last three days. And*,* I have to say*,* they do rely very much on us…[R2]*.

Nurses reported that they were the ones responsible for collecting diagnostic specimen upon doctor’s request. The majority of nurses monitored culture results and alerted the prescriber when they thought this was necessary, although they mentioned that this was not a formal nursing duty nor their responsibility.

Administering antimicrobial medication was considered an autonomous nursing activity, but communication with prescribers was frequent about the overall antimicrobial medication management, e.g. about lack of treatment effect, which triggered nurses to ask the prescriber to review the antibiotic treatment.

Nurses also frequently discussed the timing of antibiotic medication with the prescriber, mainly because of the effect of the timing of medication administration on nurses’ workflow and workload. Nurses also mentioned to be keen on prompting a switch from intravenous to oral antibiotics because it would reduce their time spent on preparing and administering intravenous antibiotics.

*….you try to reduce your workload. And if that means you can reach that goal by preparing five antibiotics less*,* you do so…. [R5]*

All nurses felt that informing the patient about the treatment was primarily the prescriber’s responsibility. On the other hand, most nurses also mentioned this as part of their role since nurses are the ones who would repeat this information in layman’s language and answer the practical, day-to-day questions about the treatment.

*……Well*,* we do tell*,* in understandable language*,* that the cultures indicate what “bug” is in the blood*,* and what antibiotic is best suited for this “bug”. And the moment an i.v. to oral switch is initiated*,* we also explain that.…….[R11]*.

Overall, nurses felt centralized in the patient care way and reflected upon themselves as “communication hub”, supporting their patient in his treatment process. Nurses ensured that patients knew what medication they were taking, along with information about potential side effects. Nurses also communicated on other aspects of patient care with the prescribers, such as wishes of the patient, but also about aspects of care that might change the direction of care or treatment. In the interdisciplinary collaboration, nurses felt they also had a guiding role when junior doctors started their rotation on the ward. They pursued them for clarification on the antimicrobial treatment, e.g. about the treatment duration. Most nurses perceived the physicians as very approachable.

#### Patient advocacy

Nurses explained their motivation to be involved in activities related to the antimicrobial treatment -as described in the previous section- as an expression of patient advocacy; striving for the best possible care for their patient.

*…In the end you’re doing this [advocating] for the patient and you want for this patient the best….[R2]*.

This motivation led them to monitor and look at culture results (an essential piece of information which could influence treatment and care decisions) and alert the prescriber on the availability of these results. Some nurses perceived this as a moral obligation, especially when their patients were clinically deteriorating. Other nurses were proactively involved in the correct ordering and documentation of antimicrobials, but always with the underlying motivation of providing the best possible care for their patient.

#### Nurse leadership

This generic competency of nurses emerged when nurses mentioned their speaking-up behavior in relation to antimicrobial treatment. They were speaking up about almost every aspect of their patients’ treatment, although some nurses felt that it was not always their responsibility. They also felt that it is a leadership trait to critically reflect on the treatment and to act upon this reflection, e.g. by asking if the antimicrobial prescription was still necessary.

*….for me nursing leadership is about optimum of care. So*,* stop what can be stopped*,* no unnecessary care*,* no unnecessary damage to your patient and being aware of what you do and why you do it to your patients. So that’s for me also nursing leadership*,* active participation in thinking about the plan. So in this sense*,* I think the responsibility or nursing leadership or antibiotic use and medication are linked to each other…. [R5]*

Nurses also mentioned that they felt it was their duty to ensure a smooth continuation of the care pathway for their patient, which motivated them to start a discussion about the treatment plan. However, they also felt this might not be their responsibility, which occasionally made them feel conflicted.

*…because on the one hand you do feel responsible for your patient*,* but on the other hand you are not the doctor’s aid*,* who keeps reminding him of new [results]…….So yes*,* it is the responsibility of the nurse to smoothen the care process………. On the other hand as a nurse*,* you cannot take responsibility for everything….[R14]*.

### Nurses’ future role

#### Enhancement of current role content

Nurses described their future participation in (appropriate) antimicrobial use in daily practice by referring to their current activities. They highlighted that their future role would be a better version of their already present competencies and activities. Nurses gave several examples on how they would shape this better version in daily practice. They felt that they could communicate more often about culture results with the prescriber and did see the benefits of this, not only for their patient but also for their own professional development.

…*and if you look at [the] waiting for culture results*,* yes*,* I think we can be more proactive…* ….*[R4]*.

Nurses also felt that other areas of nurses’ current practice could be improved. Most nurses said they could anticipate more on the i.v.-to-oral switch of antibiotics, while others believed that alertness on the presence of a stop date of the antibiotic treatment could be a shared responsibility.

Educating their patients on antimicrobial treatment was also considered an area where there was potential for improvement. Although most nurses felt that the prescriber was primarily responsible for explanation of the treatment, they also envisioned they could clarify or re-explain this information to the patient.

#### Enhancement of nursing leadership

Communicating their critical reflection on the treatment with the prescriber, “are we doing the right thing for this patient”, was considered an element that could be further developed in the future. The critical reflection and anticipation of care was also seen as part of their nursing leadership role and was shown by prompting review of the prescriber. For example, one nurse mentioned that, by prompting review of the antimicrobial treatment, prescribers would be more aware about their decision about continuation or de-escalation of antibiotic treatment.

*…So I do think that just by asking about a duration [of treatment]*,* will ensure that doctors think more consciously about it….[R14]*.

### Future role support

When asking nurses what would help them to take up this future role, their responses can be divided into three main topics: awareness and education, clarification of roles and responsibilities, and the use of cognitive reminders.

#### Awareness and education

First of all, nurses indicated that creating awareness of their role in AMS would improve their contribution. This awareness may be enhanced by clinical education which would enable them to critically reflect on matters concerning antimicrobial treatment even more.

*….Even though we know it*,* I think*,* multiple aspects*,* but awareness specifically why we give antibiotics and why it is appropriate*,* but also why we have to critically reflect to not give it too long or a too high dose or something… [R6]*.

Most nurses felt that, although sufficiently knowledgeable about administering antimicrobial treatment, more education on AMR and all matters concerning the antimicrobial treatment, would be helpful to optimize their contribution. They also felt that coaching of less experienced colleagues, e.g. nurses who were recently graduated, would help in optimizing their contribution in ward rounds. Nurses could then be better equipped to take up the nursing leadership role and actively participate and communicate in the clinical reasoning process.

Some nurses felt that it would be beneficial to promote the role of an “antibiotic champion nurse”, a nurse with expert knowledge on appropriate antibiotic use who would be easy to consult in daily practice.

#### Clarification of role and responsibilities

Nurses also felt that a clear definition of role and responsibilities would be helpful to fully exert their nursing role. Some nurses felt that this contribution to AMS was a new role for nurses and that having guidelines or a mutual agreement and commitment on the role nurses have (on a ward level) would be helpful to ensure that every nurse would attain the same AMS activities.

*….I also think that clear guidelines…….will benefit most colleagues… by installing a routine*,* to have clear guidelines when to do what*,* because otherwise it is easily forgotten…. [R4]*

Nurses also felt that the approachability of the prescriber, being open to suggestions or discussions, would be very helpful in executing their role.

#### Cognitive reminders

Ward rounds, where the treatment of patients is discussed and adjusted daily, were the most frequent opportunities for nurses to critically reflect or to prompt the prescriber to review the antimicrobial treatment. Some nurses mentioned that, during these ward rounds, the use of checklists as a participation tool could be helpful. In this way, there were made more aware of their role. Others suggested that having a reminder in the Electronic Health Record, which would prompt nurses to discuss antibiotic treatment, could be an option to increase participation.

## Discussion

This qualitative study explored nurses’ views and vision on their role regarding appropriate antimicrobial use. We identified that nurses already feel that they have a contributing role, which is grounded in the essence of nursing: patient advocacy, striving for the best possible care for their patient. Nursing leadership, a trait of this nursing role, shows itself by nurses taking action for the optimal antimicrobial treatment and care for their patient. Nurses envision their future role as an enhanced version of their current role, increasing their critical reflection and anticipation of care. Nurses perceive a shared responsibility with prescribers on certain aspects of the antimicrobial treatment and wish for a clarification of this role. Also, nurses feel they should be more aware of their role in AMS. Education and cognitive reminders such as antibiotic checklists can support the uptake of the nurses’ role.

The findings of this study are in line with the results of a recent systematic review, which states that nurses, in their daily nursing practice, are already contributing to the goals of antimicrobial stewardship [10]. Patient advocacy, as foundation of this nursing care, is described in several additional reviews [16, 17].

Interestingly, our results show that nurses considered ‘prompting review’ as an intrinsic and important element of their professional nursing leadership in daily practice. Although prompting, or questioning, the prescriber is mentioned as nursing task in the position papers and guidelines on the role of nurses in AMS [7–9, 17, 18], several studies report that nurses do not always feel this way [19–22]. Bonacansa et al. studied team interaction in surgical ward rounds in a South African hospital and observed that nurses are rarely actively engaged in antibiotic discussions [23]. A recent study in Norway, a country with a similar healthcare system as the Netherlands, Tangerees et al [19]. found that nurses did not consider prompting prescribers to review treatment as a nursing task. Our findings may be explained by the strong nursing leadership culture in the Netherlands, where speaking up about patient (safety) issues is considered a normal daily practice in the interprofessional patientcare [24, 25]. However, this nursing leadership, emphasizing clinical reasoning and anticipation of care, only flourishes when adequate knowledge and awareness is present [26]. In our study, nurses were mostly unaware of their ability to contribute to responsible antibiotic use, although they performed actions which benefitted the antimicrobial stewardship goals, e.g. suggesting intravenous to oral switch. Education of AMS principles, e.g. by using the AMS competencies framework for nurses [27], could support nurses to effectively target their already present behaviour [17, 28–30]. Cognitive reminders, such as checklists, can be implemented to assist nurses in antibiotic treatment questions, and enable nurses to fully live up to their potential [31–33].

This potential may also be helped by further clarification of the role and responsibilities of the members of the interprofessional team. Nurses in our study perceived a grey (or undefined) area of responsibilities, where they took it upon themselves to step up and, as an example, alert the prescriber on the availability of culture results (which could influence treatment decisions). Several studies acknowledge this [19, 29] and describe the need for a better definition of the nurses’ role [19, 29, 34]. This role clarification helps in interprofessional teamwork and as such is crucial to facilitate an optimal collaboration of nurses, prescriber and other healthcare professionals involved in AMS [35]. In this way, all healthcare professionals involved will provide the safest possible care for the patient [36].

### Strengths and limitations of study

The strengths of this qualitative study are that we purposefully sampled nurses from different educational backgrounds, different hospital settings geographically spread over the Netherlands, allowing a broad perspective on how nurses envision their role with regard to appropriate antibiotic use. We reached datasaturation which suggests that the full perspective of the phenomenon was captured.

A limitation of our study was the underrepresentation of nurses with a vocational background. Since leadership aspects are predominantly emphasized in the BSc curriculum [37–39], this could imply that certain aspects, e.g. nursing leadership, may be overestimated. Also, the study was conducted in hospitals in a single high-income country with a specific universal healthcare system [40] with a strong emphasis on patient safety [41, 42], which may influence the way nurses view and execute their role and activities.

### Implications for the future of nurses’ contribution in antimicrobial stewardship

The results of this qualitative study may further guide strategies to strengthen the bedside nurses nursing contribution to AMS goals, e.g. by referring to motivational factors of the nursing contribution (e.g. patient advocacy) or by developing nurse-specific education (e.g. coaching on speak-up competencies related to antimicrobial treatment). We summarized our recommendations with regard to policies, professionals and practice, in Table 2.

**Table 2:** Recommendations to support bedside nurses’ role

Policies	• Emphasize and document in national guidelines for Antimicrobial Stewardship, that the multidisciplinary team collaboration at the bedside (physicians, pharmacists, medical microbiologist, nurses and other allied healthcare professionals), is key to provide the most optimal antimicrobial treatment • Describe in the national guidelines for Antimicrobial Stewardship, the specific role bedside nurses have in medication optimization and appropriate antibiotic use • Acknowledge and document in the local Antimicrobial Stewardship guidelines the role bedside nurses, as members of the interprofessional healthcare team providing bedside care, can have in medication optimization and appropriate antibiotic use.
Professional	• Implement the content of the WHO “Curriculum guide on Health workers’ education and training on antimicrobial resistance” in both pre- and post-licensure education for nurses [43]. • Ensure bedside nurses have a solid foundation of knowledge and skills on the following topics: • AMR/AMS, Antimicrobial treatment and related matters (clinical reasoning for nurses, diagnostics, patient education), interprofessional communication/collaboration and nursing leadership [44, 45] • Tailor the teaching method and content to the educational needs of the targeted bedside nurses [46] • Tailor the teaching method to the local setting and influencing circumstances (e.g. workload, team culture) [46] • Make use of “coaching-on-the job” and “role modelling” as strong educational modes [47] • Support interprofessional teamwork: o Encourage team communication on values and goals [48] o Clarify roles and contributions of the individual team members [48] o Emphasize that respectful collaboration and teamwork is essential for providing high quality of care [49]
Practice	• Make use of and implement cognitive reminders in the daily nursing practice. Examples of cognitive reminders are checklists (to be used during ward rounds) or reminders in the Electronic Health Care system. • Appoint an “Antibiotic Champion”, a nurse with extended knowledge and skills regarding Antimicrobial Stewardship, at the ward level • Establish a formal partnership between either the dedicated bedside nurse (Antibiotic Champion) or a representative of the bedside nurses, with a member of the Antimicrobial Stewardship Team at the hospital level. The goal of the partnership is to support the bedside nurses in daily practice but also to work together on quality improvement projects related to AMS.

## Conclusion

Dutch hospital-based nurses feel that they are already contributing to appropriate antimicrobial use in their patients. They envision their future role as an enhanced, elaborated and empowered version of their current daily practice. Further development of this role may be supported by increased role-awareness, education on AMR/AMS in general and further clarification of the (sometimes shared) responsibilities of both nurses and prescribers.

### Electronic supplementary material

Below is the link to the electronic supplementary material.

Supplementary Material 1

## Data Availability

All data are in native language. Data may be shared upon reasonable request if individual consent has been granted by the participant.
