# Supplementary material for: Nurses’ contribution to antimicrobial stewardship: business as usual?
Source: Antimicrob Resist Infect Control. 2024 Aug 29;13:93. doi: 10.1186/s13756-024-01451-z (PMC11361204; doi:10.1186/s13756-024-01451-z)
Supplement: Supplementary file 1 — Supplementary Material 1 [file 13756_2024_1451_MOESM1_ESM.docx]

**Supplementary file 1 Topiclist**

1. Envision a patient with a potential infection, who is admitted to the ward where you work, who potentially will receive antibiotics.
   1. What is your role with regard to this?
   2. How does your professional conduct influence appropriate antibiotic use?
2. How do you envision the role of the bedside nurse in relation to appropriate or responsible use of antibiotics?
3. How can the (future) role of the bedside nurse be supported?
